# CXCL13 and CXCL9 CSF Levels in Central Nervous System Lymphoma—Diagnostic, Therapeutic, and Prognostic Relevance

**DOI:** 10.3389/fneur.2021.654543

**Published:** 2021-03-26

**Authors:** Ilias Masouris, Kirsi Manz, Markus Pfirrmann, Martin Dreyling, Barbara Angele, Andreas Straube, Sigrid Langer, Marion Huber, Uwe Koedel, Louisa Von Baumgarten

**Affiliations:** ^1^Department of Neurology, University Hospital, Ludwig Maximilian University, Munich, Germany; ^2^Institute for Medical Information Processing, Biometry, and Epidemiology, Ludwig Maximilian University, Munich, Germany; ^3^Department of Medicine III, Ludwig Maximilian University, Munich, Germany; ^4^Max Planck Institute of Psychiatry, Munich, Germany; ^5^Department of Neurosurgery, University Hospital, Ludwig Maximilian University, Munich, Germany

**Keywords:** CNS lymphoma, cerebrospical fluid, biomarker, CXCL13 chemokine, CXCL9

## Abstract

**Background:** Diagnostic delay and neurologic deterioration are still a problem for the treatment of rapidly progressing CNS lymphoma (CNSL); there is an unmet need for a diagnostic test with a high diagnostic yield and limited risk, minimizing the time to the initiation of effective treatment.

**Methods:** In this prospective monocentric study, we analyzed the utility of CXCL13 and CXCL9 as diagnostic, therapeutic and prognostic biomarkers for CNSL. Cerebrospinal fluid (CSF) from 155 consecutive patients admitted with brain lesions of various origins was collected. Levels of CXCL13 and CXCL9 were analyzed by ELISA. Additionally, CSF was analyzed during CNSL disease course (relapse, remission, progress) in 17 patients.

**Results:** CXCL13 and CXCL9 CSF levels were significantly increased in patients with CNSL compared to control patients with lesions of other origin. Using logistic regression and a minimal-*p*-value approach, a cut-off value of 80 pg/ml for CXCL13 shows high sensitivity (90.7%) and specificity (90.1%) for the diagnosis of active CNSL. CXCL9 at a cut-off value of 84 pg/ml is less sensitive (61.5%) and specific (87.1%). Both cytokines correlate with the clinical course and response to therapy.

**Conclusions:** Our results confirm the excellent diagnostic potential of CXCL13 and introduce CXCL9 as a novel albeit less powerful marker for PCNSL.

## Introduction

Central nervous system lymphoma (CNSL) accounts for ~1–5% of all brain tumors and comprises two entities, primary and secondary CNSL (PCNSL, SCNSL) (1). PCNSL represents a lymphoma of the diffuse large B cell subtype exclusively confined to the CNS at the time of diagnosis (2). Secondary CNS involvement occurs in ~5% of patients with systemic lymphoma (1). With MTX-based chemotherapy, long-term survival can be achieved for up to one third of the CNSL patients (3). Nonetheless, for most patients, prognosis remains dismal with a median survival of 26 months (4). Without therapy, CNSL can be fatal within 2–3 months.

The rapidly progressive nature of CNSL requires a timely diagnosis and prompt initiation of therapy. However, diagnostic delay is still a major problem (5). MRI alone does not allow a reliable distinction from other CNS lesions of neoplastic, inflammatory, or infectious origin (6, 7). In <15%, positive cerebrospinal fluid (CSF) or vitreous biopsy for lymphoma can eliminate the need for histopathologic confirmation by brain biopsy (8). However, brain biopsy is required to diagnose the vast majority of CNSL patients. Nevertheless, brain biopsies have a potential risk of complications including hemorrhage, infection, and non-diagnosis (9). Especially treatment with steroids prior to biopsy increases the risk of diagnostic failure in up to >50% of cases (10). Furthermore, some lesions are not amenable to biopsy because of their small size, location in deep brain structures, or the risk of hemorrhage. Therefore, there is a need for alternative diagnostic tests with high diagnostic yield and limited risks, leading to shorter time to diagnosis and rapid treatment.

In recent years, several candidate molecules in blood and CSF have been identified that might be useful as diagnostic biomarkers for CNSL (11–16). Among these, especially CXCL13 has a high potential as a diagnostic marker for CNSL, as evidenced by a recent meta-analysis (17). CXCL13 has been shown to be upregulated in CSF of patients with CNSL, its levels decrease under therapy and have been shown to be negatively correlated with patient survival (18, 19). Overall, CSF-biomarkers have a great potential as a non-invasive diagnostic tool in CNSL. However, their diagnostic and prognostic accuracy has not been studied in a prospective cohort of patients.

The goal of this study was to identify relevant diagnostic markers in the CSF of patients with PCNSL and to validate their diagnostic potential in a prospective setting in a monocentric study.

## Materials and Methods

### Patients

This prospective study was conducted in the University Hospital of Munich, Germany from 2012 to 2015. All patient samples were collected following written informed consent according to local ethics guidelines and the Declaration of Helsinki. We included consecutive patients > 18 years with at least one MRI-proven brain lesion of unknown origin, in whom diagnostic lumbar puncture was performed during clinical routine. Diagnosis of CNSL was established by stereotactic brain biopsy and/or by CSF analysis. Clinical data, radiographic data, and laboratory results were obtained. Two prognostic scores [the International Extranodal Lymphoma Study Group (IELSG) score (20) and the Memorial Sloan-Kettering Cancer Center (MSKCC) score (21)] were determined.

### Processing of CSF and Serum Samples

CSF and serum samples were collected, immediately centrifuged, and stored at −80°C. Routine CSF analysis (i.e., cell count, microscopy, protein quantification, glucose levels) as well as cytology, immunophenotyping, and flow cytometry were performed at our Institute of Laboratory Medicine.

### Protein-Array

To identify potential markers for CNSL, archived CSF samples from patients with untreated PCNSL (new diagnosis/ND and relapse/R, *n* = 5, respectively) were analyzed for levels of various cytokines and chemokines (see Supplemental Material) using a custom-made protein array. CSF samples from patients with tension headache, CNS metastasis with leptomeningeal involvement, and primary brain tumors (*n* = 5, respectively) were used as controls. The array was applied following the instructions of the manufacturer (Raybiotech Inc., USA).

### ELISA

Levels of soluble CXCL13 and CXCL9 in the CSF were determined using ELISA (R&D Systems) following the manufacturer's instructions.

### Magnetic Resonance Imaging

MRI analysis included a T1-weighted, a T1 weighted contrast enhanced, a T2-weighted, a diffusion weighted (DWI) and a fluid-attenuated inversion recovery weighted (FLAIR) sequence. CXCL13 and CXCL9 levels were compared for patients with different CNSL MRI-characteristics: (1) homogenous vs. heterogenous contrast enhancement (2) contact vs. no contact to ventricular system (3) involvement vs. no involvement of deep brain structures, and (4) monolocular vs. multilocular occurance. MRI evaluation was performed as central review by an experienced neuro-oncologist (MH).

### Statistics

For the comparison of a continuous variable between two groups, we applied the Mann Whitney *U* test for independent groups and the Wilcoxon test for dependent groups. The association of two categorical variables was assessed using the chi-square test. Correlation between two continuous variables was investigated with Spearman's correlation coefficient. Unless otherwise indicated, values of continuous variables were described by median and range.

Receiver operating characteristic (ROC) curves and the area under the curve (AUC) were calculated for the biomarkers. Youden's index was used to choose the optimal cut-off.

To investigate factors associated with the diagnosis of CNSL, logistic regression was applied. Candidate prognostic factors for multiple regression analyses were age at diagnosis, sex, CXCL9, and CXCL13 CSF levels. For continuous variables, the potential inclusion of a first-degree fractional polynomial was considered as alternative to fitting a straight line (22).

To aid in medical decision making, the linear predictor of the final model was intended to be categorized into prognostic groups. The classification was done with the minimum *p*-value approach, with adjustment for multiple testing and assuming that the smallest group should contain at least 20% of patients (23). Bootstrap resampling was applied to choose the final model and to assess the stability of classifications (24).

Disease-free survival probabilities were estimated by the Kaplan-Meier method; different groups were compared using the log-rank test. Cox regression was applied to estimate hazard ratios.

Apart from *p*-value adjustment for multiple testing, the significance level of the two-sided *p*-values was 0.05. Estimates were given with 95% confidence intervals. All analyses were exploratory. Analyses were done with SAS version 9.4, an SAS macro for multiple fractional polynomials, the statistics software R version 3.4.3, the Prism Software, or SigmaPlot.

## Results

In a first step, we aimed to identify relevant diagnostic biomarkers for CNSL. Out of 35 candidate cytokines, we identified 3 promising biomarkers (Supplementary Figure 1) using a cytokine/chemokine array. CXCL13 and IL10, which have been previously described as being elevated in patients with CNSL (18, 19, 25), and CXCL9, which has not been reported as a biomarker for CNSL so far. Since CXCL13 has robustly been shown to have a high sensitivity and specificity, we chose to confirm its diagnostic potential in a prospective setting. Furthermore, CXCL9, a biomarker not described as a CSF biomarker so far, was analyzed for its diagnostic potential.

One hundred and fifty-five consecutive patients with brain lesions of various origins were included in our study: 46 patients with PCNSL, 8 patients with SCNSL, 24 patients with primary brain tumors (PBT), 23 patients with secondary brain tumors (SBT), 7 patients with lesions from neuroinfectious diseases (NID), 29 patients with lesions from autoimmune diseases (AID), and 18 patients with focal lesions from other neurological diseases (OND). All CNSL specimen were classified as diffuse large B cell lymphoma by histopathologic analysis. Patient characteristics and respective CSF parameters are shown in Table 1 and a detailed description of the respective diagnoses can be found in the Supplementary Material.

**Table 1:** Patient characteristics and cerebrospinal fluid (CSF) parameters for the different patient groups.

	**Patient characteristics**			**CSF parameters**			
**Diagnosis**	***n***	**Age**	**Sex** ** (f/m)**	**Cells/μl**	**Protein** ** (mg/dl)**	**Glucose** ** (mg/dl)**	**AI CSF/** **serum (× 10^**−3**^)**
Newly diagnosed PCNSL	32	52 (21–85)	14/18	7 (1–408)	66 (9–604)	64 (41–93)	10 (4–96)
Newly diagnosed SCNSL	8	67 (53–90)	3/5	7 (2–369)	89 (50–221)	53 (34–103)	13 (8–53)
Relapsed PCNSL	14	64 (23–81)	6/8	8 (1–202)	88 (39–448)	63 (25–99)	18 (3–65)
PCNSL complete remission	22	70 (31–80)	9/13	1 (0–8)	54 (30–204)	65 (46–112)	9 (4–49)
Primary brain tumor (PBT)	24	60 (21–83)	8/16	2 (0–204)	57 (25–193)	60 (48–89)	8 (3–50)
Secondary brain tumor (SBT)	23	63 (31–83)	11/12	5 (0–165)	65 (26–336)	63 (10–98)	7 (4–55)
Autoimmune inflammatory disease (AID)	29	48 (19–76)	19/10	9 (0–144)	54 (23–128)	60 (25–76)	7 (3–17)
Neuroinfectious disease (NID)	7	58 (20–76)	1/6	16 (1–596)	64 (49–104)	54 (36–65)	12 (7–13)
Other neurologic disease (OND)	18	57 (30–84)	12/6	2 (0–7)	45 (20–130)	65 (55–116)	6 (2–21)
Historical non-lesional controls	101	53 (18–87)	48/53	1 (0–13)	44 (22–292)	62 (45–104)	6 (2–16)

*For age, cells, protein and glucose levels and antibody index, median with range was calculated. n, number of patients of each group; f, females; m, males; AI, albumin index*.

To rule out the possibility that patients without focal CNS lesions harbor elevated CXCL13 or CXCL9 CSF levels, we also used archived CSF samples from 101 patients with various non-lesional neurological diseases as historical controls (for diagnoses, see Supplementary Material).

### CXCL13 and CXCL9 CSF Levels Are Elevated in Active CNSL

**Figure 1:**
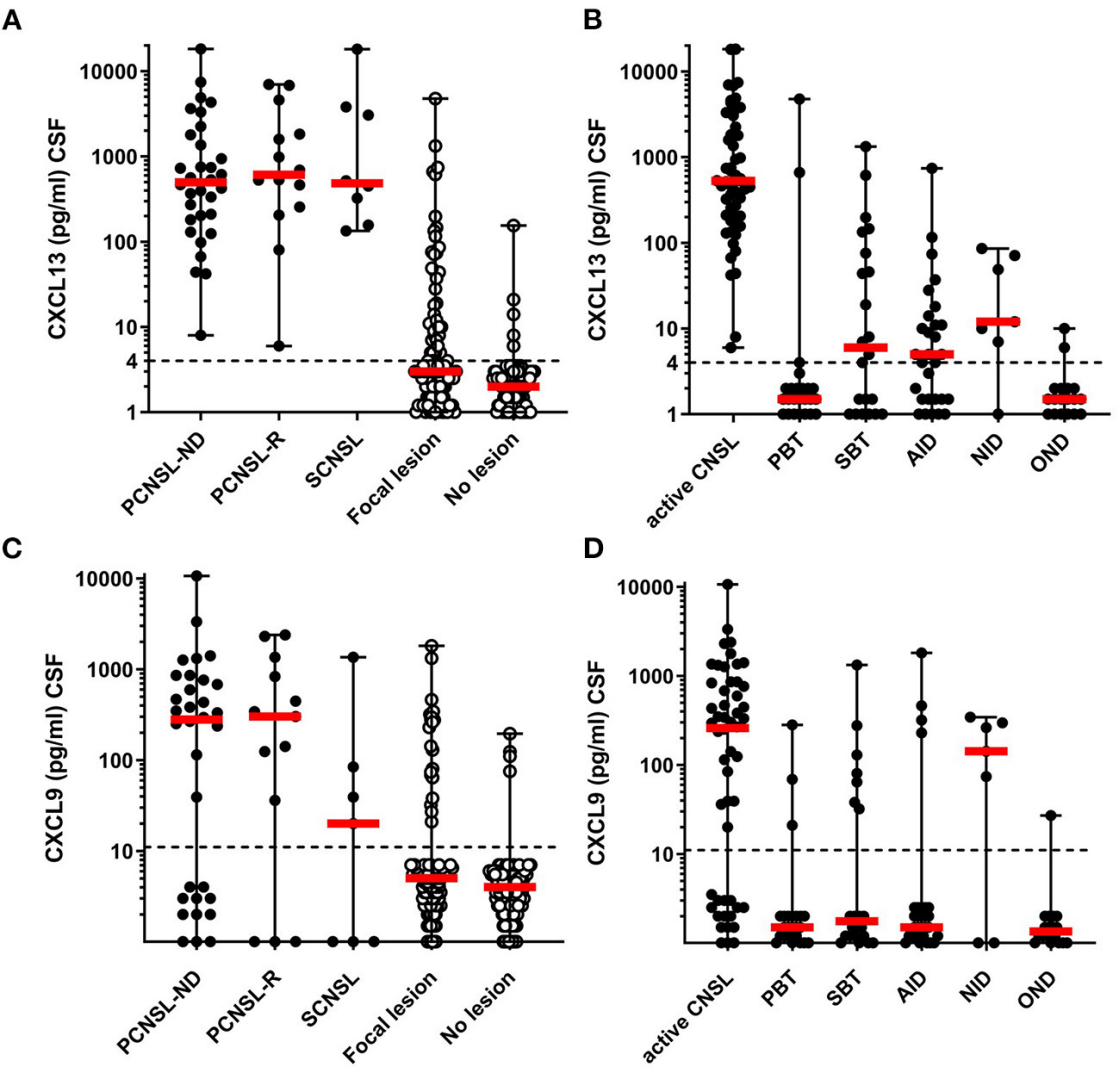
CXCL13 and CXCL9 levels in CNSL and other lesions. CXCL13 CSF levels **(A,B)**, and CXCL9 CSF levels **(C,D)** were determined in samples for patients with CNSL and other lesional brain pathologies. **(A,C)** CSF levels of CXCL13 and CXCL9 were compared between patients with newly-diagnosed primary CNSL (PCNSL-ND), PCNSL in relapse (PCNSL-R), secondary CNSL (SCNSL), and all lesional pathologies grouped together (Focal lesions) as well as patients with other non-lesional pathologies serving as negative controls (No lesion). **(B,D)** CXCL13 and CXCL9 levels were compared between all patients with active CNSL and those with other lesional pathologies subcategorized into primary brain tumors (PBT), secondary brain tumors (SBT), autoimmune neuroinflammatory diseases (AID), neuroinfectious diseases (NID), and other lesions (OND). The dotted line in each diagram depicts the detection limit of each chemokine. Values below the detection limit were counted as 0 for calculation processes but were assigned different values in the diagram for better visualization of the number of probes below the limit.

CSF CXCL13 levels of patients with active (untreated) primary and secondary CNSL at diagnosis or relapse did not indicate a clinically relevant difference [PCNSL-ND 499 (8–18,292), *n* = 32; PCNSL-R 613 (6–7,007), *n* = 14; SCNSL 485 (134–18,126) pg/ml, *n* = 8; *p* = 0,774, Figure 1A], and were therefore pooled for further analysis. CSF levels of CXCL13 of patients with active CNSL were significantly higher compared with patients with focal lesions of other origin [0 (0–4,789) pg/ml, *n* = 101, *p* < 0.001] or with historic controls without CNS lesion [0 (0–155) pg/ml, *n* = 101, *p* < 0.001] (Figure 1A).

Subgroup analysis of all patients with focal lesions revealed that CXCL13 CSF levels were significantly higher in patients with active CNSL [530 (6–18,292) pg/ml] as compared to PBT [0 (0–4789) pg/ml], SBT [7 (0–1,333) pg/ml], AID [5 (0–744) pg/ml], NID [12 (0–86) pg/ml], and other CNS lesions [OND 0 (0–10) pg/ml] (Figure 1B).

CXCL9 CSF levels were elevated in patients with active CNSL with no significant difference between the subgroups {PCNSL-ND [280 (0–10,676)], PCNSL-R [342 (0–2,379)], SCNSL [20 (0–1,354) pg/ml], *p* = 0.165}; thus we pooled these samples (active CNSL) for further analysis (Figure 1C).

Subgroup analysis revealed significantly higher CXCL9 CSF levels in patients with active CNSL [259 (0–10,676) pg/ml] as compared to patients with PBT [0 (0–282) pg/ml, *p* < 0.001], SBT [0 (0–1,326) pg/ml, *p* = 0.002], AID [0 (0–1,808) pg/ml, *p* < 0.001], and OND [0 (0–27) pg/ml, *p* < 0.001]. CXCL9 CSF levels of patients with lesions from NID, however, did not significantly differ from those of patients with CNSL [142 (0–343) pg/ml] (Figure 1D).

In a next step, we analyzed whether CXCL13 and CXCL9 CSF levels in patients with active CNSL are associated with patient characteristics (age, gender), prognostic scores (IELSG, MSKCC), MRI- and CSF characteristics (protein level, leucocyte count, cell count, glucose levels, albumin ratio, IgG ratio, meningeosis lymphomatosa; data not shown). We found a weak to moderate positive correlation of CXCL13 CSF levels with CSF leukocyte count (Spearman correlation coefficient rho = 0.48), CSF protein levels (rho = 0.47), albumin ratio (rho = 0.58), and IgG ratio (rho = 0.53). Moreover, CXCL13 levels were significantly higher in patients with meningeosis lymphomatosa as compared to those without (*p* = 0.043). CXCL9 levels showed a weak to moderate positive correlation with CSF protein levels (rho=0.53), albumin ratio (rho = 0.61), and IgG ratio (rho = 0.63). Both markers correlated positively with each other (rho = 0.66). Furthermore, we compared the CSF levels of CXCL13 and CXCL9 in patients with different MRI characteristics. Neither CNSL contact to the ventricular system, nor homogenous contrast enhancement, involvement of deep brains structures or mulitlocular occurance were associated with increased CXCL13 or CXCL9 levels.

Since steroids can induce rapid regression of CNSL we compared the CSF CXCL13 and CXCL9 levels of patients with or without current or previous steroid treatment. Information on the steroid medication could be obtained in 51/54 patients with CNSL. Of those, 12 patients received steroids, 39 patients did not. CXCL13 CSF levels were substantially lower in patients with steroid intake [157.5 (6–18,292) pg/ml] than in those without [616 (80–18,126) pg/ml], however, the difference was not statistically significant (*p* = 0.077). Similarly, CXCL9 CSF levels in patients treated with steroids were also lower, the difference did not reach statistical significance [114 (0–10,676) pg/ml vs. 266 (0–3,347) pg/ml, *p* = 0.776].

### Diagnostic Potential of CXCL13 and CXCL9

**Figure 2:**
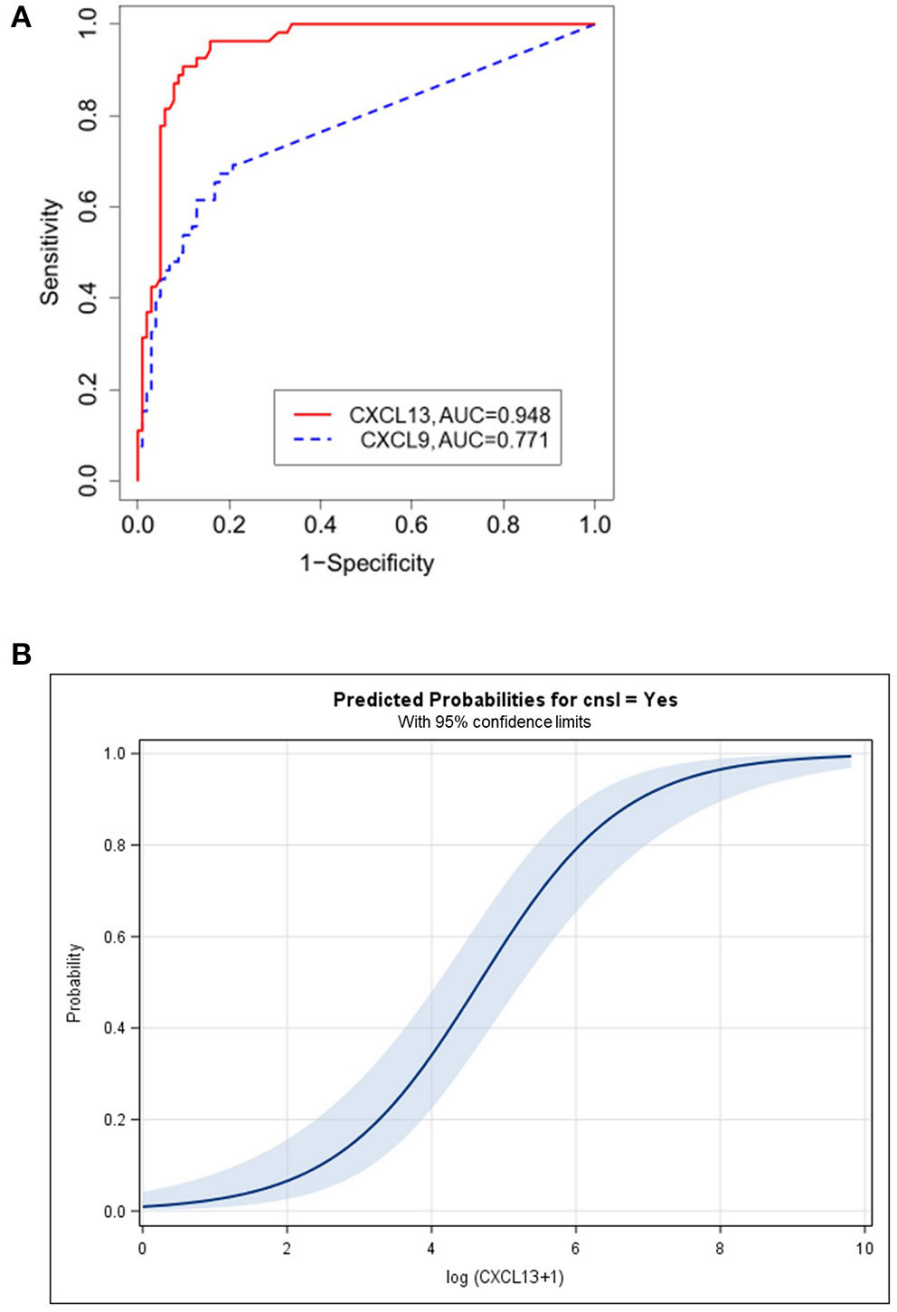
**(A)** Diagnostic potential of CXCL13 and CXCL9. ROC-analysis of all 155 patients with cerebral focal lesions for CXCL13 (red line) and CXCL9 (blue line). AUC, Area under curve. **(B)** Probabilities for CNSL predicted by the logistic regression model. Each individual CXCL13 CSF level was transformed to LOGC, logarithm of [CXCL13 (pg/ml) + 1]. For each transformed LOGC, the linear predictor (LP) of the logistic regression model was calculated: LP = −4.649 + 0.997 × LOGC. Finally, the probability of CNSL was estimated by *p* = 1 / [1 + exp(−LP)]. CNSL, CNS lymphoma; CSF, Cerebrospinal fluid.

To evaluate the diagnostic potential of CXCL13 and CXCL9, we assessed their diagnostic sensitivity and specificity. Respective ROC curves are shown in Figure 2A. For CXCL13, the area under the ROC curve (AUC) was 0.948 [95% confidence interval (CI): 0.914; 0.982]. For CXCL9 it was 0.771 (0.693; 0.848).

Based on Youden's index, we chose a diagnostic cut-off CXCL13 CSF level of ≥80 pg/ml and a CXCL9-level of ≥36 pg/ml. At these cut-off levels, we found a sensitivity of 90.7% (79.7; 96.9%), a specificity of 90.1% (82.5; 95.2%), and an OR of 89.18 (26.24; 335.86, *p* < 0.0001) for CXCL13. For CXCL9, the sensitivity of 67.3% (52.9; 79.7%) and specificity of 82.2% (73.3; 89.1%) as well as the OR 9.49 (4.11; 22.12, *p* < 0.0001) were lower.

To further verify our approach in a more sophisticated statistical strategy, we applied the minimal *p*-value approach. Using this strategy, 80 pg/ml was confirmed as an ideal cut-off for CXCL13. However, for CXCL9, 84 pg/ml (< vs. ≥) was a slightly better alternative [sensitivity 61.5% (47.0; 74.7%), specificity 87.1% (79.0; 93.0%), with maximum OR of 10.83 (95% CI: 4.51; 26.42, *p* < 0.0001)]. In bootstrap resampling, 84 pg/ml was most often chosen leading to less misclassified patients. Table 2 summarizes the sensitivity, specificity and odds ratio values of the two chemokines.

**Table 2:** Sensitivity, specificity and odds ratio for CXCL13 and CXCL9 in CNSL.

**Marker**	**Cut-off** **-value**	**Sensitivity** ** (%)**	**Specificity** ** (%)**	**Odds ratio**	**AUC**	***p*-value**
CXCL13	80 pg/ml	90.7% (79.7; 96.9%)	90.1% (82.5; 95.1%)	89.2 (26.2; 335.9)	0.948 (0.914; 0.982)	*p* < 0.0001
CXCL9	36 pg/ml	67.3% (52.9; 79.7%)	82.2% (73.3; 89.1%)	9.5 (4.1; 22.1)	0.771 (0.693; 0.848)	*p* < 0.0001
	84 pg/ml	61.5% (47.0; 74.7%)	87.1% (79.0; 93.0%)	10.8 (4.5; 26.4)	0.771 (0.693; 0.848)	*p* < 0.0001

CXCL13 levels were elevated above the predetermined cut-off of 80 pg/ml in 10 patients without CNSL: 2 patients with PBT (glioblastoma); 5 patients with SBT and concomitant meningeosis carcinomatosa deriving from breast (3) and non-small cell lung cancer (NSCLC) (2); 2 AID patients (multiple sclerosis); and 1 NID patient (septic emboli from bacterial endocarditis and concomitant meningitis). CXCL9 was elevated above the threshold of 84 pg/ml in 12 patients without CNSL: 1 patient with PBT (glioblastoma); 3 patients with SBT from NSCLC (2) and melanoma (1); 4 AID patients (2 multiple sclerosis, 1 neurosarcoidosis, 1 cerebral vasculitis); and 4 NID patients (1 progressive multifocal leukoencephalopathy, 1 cerebral toxoplasmosis, 2 aspergillomas). One patient with multiple sclerosis and one patient with NSCLC and cerebral metastases showed levels above the cut-off value for both proteins. Five patients with active CNSL were below the cut-off value of 80 pg/ml for CXCL13. Interestingly, all of them were under steroid medication.

### Diagnostic Potential of CXCL13 and CXCL9—Logistic Regression Analysis

To further validate the diagnostic potential of CXCL13 and CXL9, a logistic regression analysis was performed. CXCL13 outperformed the other prognostic variables (CXCL9, age, sex) included in this model. With this respect, CXCL13 was always included in 1,000 bootstrap samples when the best multiple model was determined. In 99.2% of the cases, CXCL13 was modeled as a first-degree fractional polynomial [predominantly either “1/square root (CXCL13 + 1)” or “log (CXCL13 + 1)”]. Sex, CXCL9 (or any transformation), and age were rarely included (only in 18.9, 6.0, and 36% of the cases respectively).

Besides CXCL13 [described as log (CXCL13 + 1)], no further variable was significant. The odds ratio for log (CXCL13 + 1) was 2.71 (95% CI: 1.99; 3.69). Therefore, the logistic regression model allowed the probabilities for CNSL for individual CXCL13 levels to be predicted, as highlighted in Figure 2B. Applying the transformation log (CXCL13 + 1) to the CXCL13 CSF cut-off 80 pg/ml results in 4.39, with a predicted probability of 43.3% (95% CI: 30.5; 57.0%). In the logistic model, the estimate of the intercept was−4.649 (95% CI: −6.417; −3.332) and the regression coefficient for log (CXCL13 + 1) was 0.997 (95% CI: 0.727; 1.352). The values of log (CXCL13 + 1) were grouped into seven groups (0, >0–2, >2–3, …, >7, see Table 3). Within each group, the observed proportion of patients with CNSL was calculated (column 6) together with the corresponding 95% confidence interval. For the CXCL13 value of each patient the expected proportion of CNSL was calculated (Step 1 to 3, Table 3). Within the patients of each group, the mean expected proportion of CNSL was estimated. The means of all expected proportions lay within the 95% CI around the observed proportions.

**Table 3:** Probabilities for CNSL calculated from logistic regression in dependence on CXCL13 CSF levels.

**Groups defined** ** by log [CXCL13** ** (pg/ml) + 1]** ** values**	**Original** ** CXCL13** ** values** ** (pg/ml)^§^**	**Mean of estimated** ** probabilities of** ** CNSL (%)^¶^**	**Total number** ** of patients** ** (*N*)**	**Patients with** ** CNSL** ** (*N*)**	**Observed group** ** proportion with** ** CNSL (%)**	**95% confidence** ** interval for observed** ** proportion (%)^$^**
0	0	0.9	54	0	0	0-6.7
>0–2	1–6	4.4	15	1	6.7	1.2-29.8
>2–3	7–19	10.2	16	1	6.3	1.1-28.3
>3–4.3	28–76	33.0	11	3	27.3	9.8-56.6
4.4–6	80–396	62.2	21	16	76.2	54.9-89.4
>6–7	425–987	84.9	19	16	84.2	62.4-94.5
>7	1,333–18,292	96.7	19	17	89.5	68.6-97.1

*CNSL, CNS lymphoma; CSF, Cerebrospinal fluid*.

§ *The ranges of the values correspond to the values actually observed in the groups, with the groups defined by the logarithm in column 1. The logarithm is always the natural logarithm to the base of the mathematical constant*.

¶ *Within each group, for each patient the probability of CNSL was estimated from the individual CXCL13 CSF level using the logistic regression model. Group intervals were defined by log [CXCL13 (pg/ml) + 1]*.

Step 1: Each CXCL CSF value was transformed: LOGC, logarithm of [CXCL13 (pg/ml) + 1]. Step 2: For each transformed LOGC, the linear predictor (LP) of the logistic regression model was calculated: LP = −4.649 + 0.997 × LOGC. Step 3: The probability of CNSL was estimated by p = 1/[1 + exp(-LP)]. Step 4: The mean of all probabilities in each group was calculated.

$ *Confidence intervals in accordance with Wilson*.

### CXCL13 and CXCL9 as Parameters of Disease Activity

**Figure 3:**
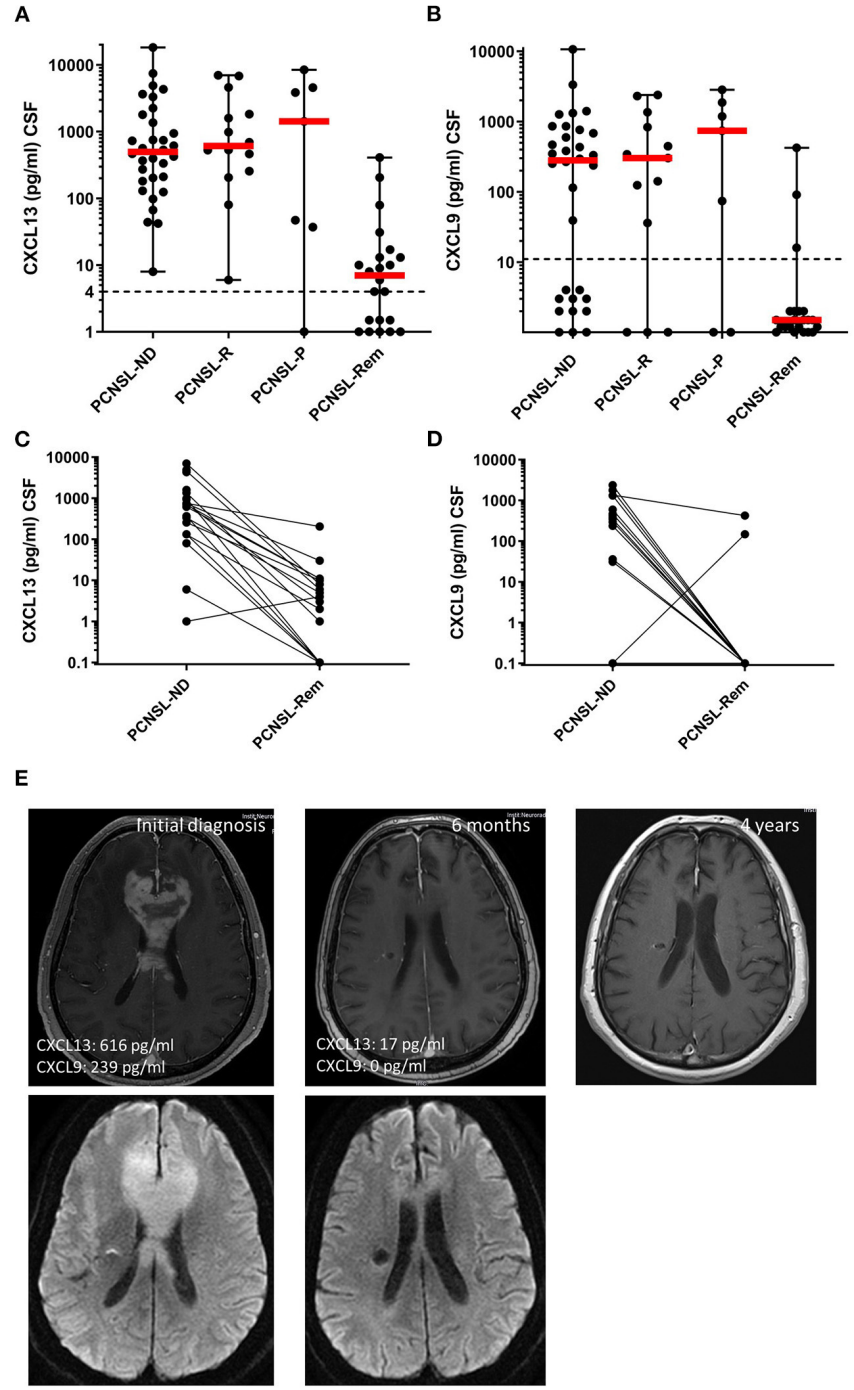
CXCL13 and CXCL9 as disease monitoring and prognostic markers. **(A,B)** CXCL13 and CXCL9 CSF levels were compared between patients with newly-diagnosed PCNSL (PCNSL-ND), PCNSL in relapse (PCNSL-R), PCNSL with disease progress during therapy (PCNSL-P), and PCNSL in remission (PCNSL-Rem). The dotted line in each diagram depicts the detection limit of each chemokine. Values below the detection limit were counted as 0 for calculation processes but were assigned different values in the diagram for better visualization of the number of probes below the limit. **(C,D)** CXCL13 and CXCL9 CSF levels were compared in patients with PCNSL before and after 6 cycles of chemotherapy, where the disease was declared to be in remission by MRI and CSF withdrawal. **(E)** MRI images of a patient with CNSL upon initial diagnosis (left), remission after 6 cycles of chemotherapy (middle), and long-term remission 3 years later (right).

In order to assess the potential of CXCL13 and CXCL9 to monitor disease activity, their CSF levels were compared in (1) active CNSL (*n* = 54), (2) in patients who showed disease progress under chemotherapy (PCNSL-P, *n* = 7), and (3) in patients with complete remission after therapy (PCNSL-Rem, *n* = 22). Compared to patients with active CNSL, CSF levels of CXCL13 and CXCL9 were significantly reduced in patients in complete remission (Figures 3A,B).

Seventeen patients with active PCNSL were analyzed before and after the completion of 6 cycles of MTX-based polychemotherapy, all of them had confirmed complete remission (Figures 3C,D). Both chemokines significantly decreased after therapy: CXCL13 CSF levels dropped from 616 (1–7,007) pg/ml to 4 (0–205) pg/ml (*p* = 0.0054), CXCL9 CSF levels decreased from 269 (0–2,379) pg/ml to 0 (0–423) pg/ml (*p* < 0.0001). Figure 3E shows MRI images of the CNSL brain lesion of a representative patient upon initial diagnosis until after remission.

In a final step, the potential of the two chemokines as prognostic biomarkers was assessed. Of the 46 patients with active PCNSL who were included in our study, survival data was obtained from 39 patients. We analyzed relapse-free survival during a follow-up period of 2 years. The minimal *p*-value approach did not reveal any stable cut-off for either chemokine (data not shown). Since a cut-off for CXCL13 at 200 pg/ml has been related to inferior outcome previously (18), it was applied in our data set to explore its performance (Supplementary Figure 2). We did not find a significant difference in the relapse-free survival between patients with CXCL13 values above and below the cut-off [hazard ratio HR = 1.92 (0.72–5.12), *p* = 0.184]. The corresponding sensitivity and specificity for this cut-off were 77.8 and 95.0%, respectively.

## Discussion

Diagnostic delay and concomitant neurologic deterioration are still a problem for CNSL patients and there is an unmet need for diagnostic tests with a high diagnostic yield and limited risk, minimizing the time until initiation of treatment. Here we report the results of a prospective, monocentric study on the potential of CXCL13 and CXCL9 as CSF biomarkers for CNSL. We could show that both proteins are significantly elevated in the CSF of patients with CNSL when compared with patients with CNS lesions of other origin. Detailed analysis revealed that CXCL13 has a superior diagnostic potential and the additional analysis of CXCL9 showed no additional benefit. Interestingly, both markers dropped in response to PCNSL therapy. Furthermore, at relapse, CSF levels were significantly elevated, indicating that serial assessment may allow disease monitoring.

CXCL13 is crucial for the homing and motility of B cells in lymphoid tissue and has been implicated in the formation of ectopic lymphoid tissue in chronic inflammation and cancer (26, 27). CXCL13 expression by malignant B cells in CNSL was first reported by Smith et al. (28) and was since reproduced by other groups (29–31). To date, four studies analyzed CXCL13 CSF levels as biomarkers in CNS lymphoma. A retrospective study compared CSF CXCL13 levels of 30 patients with CNSL patients to 40 control patients with and without other CNS malignancies (32). Significantly higher CSF CXCL13 levels were documented in patients with CNSL compared to control patients; however, no data on the diagnostic accuracy was reported. A second retrospective study also showed significantly higher CSF CXCL13 levels in patients with CNSL and an excellent diagnostic accuracy (AUC: 0.981), however no data on sensitivity/specificity and cut-off value were reported (33). Rubenstein et al. has published a large, multicentric retrospective study on CSF CXCL13 and CSF IL10 levels. 220 patients were included (83 patients with CNSL and 137 relevant disease controls). The authors report a sensitivity of 69.9% and a specificity of 92.7% in the discrimination of CNSL for an increased CSF CXCL13 level > 90 pg/ml (18). A monocentric retrospective study analyzed the combined diagnostic performance of CSF CXCL13, CSF IL-10 and the apparent diffusion coefficient (ADC) on cerebral magnetic resonance imaging (cMRI) for 43 CNSL and 44 relevant disease controls (19). A sensitivity of 76.7% and a specificity of 90.9% was reported for CSF CXCL13 at a cut-off value of >103 pg/ml. In our study, we could further substantiate the diagnostic utility of elevated CXCL13 CSF levels in a prospective consecutive setting. Based on our data, a cut-off level of 80 pg/ml was used, which, with a considerable sensitivity of 90.7% and specificity of 90.1%, discriminates CNSL from cerebral lesions of infectious, inflammatory, and malignant origin. Investigating the logarithmic transformation of CXCL13 CSF levels in a logistic regression model, we found that 80 pg/ml defined a real turning point (Figure 3 and Table 3). With values lower than the cut-off, the proportion of patients with CNSL were distinctively lower than in groups with CXCL13 CSF levels higher than the cut-off.

However, elevated CSF levels of CXCL13 have been described for certain neuroinfectious conditions such as neuroborreliosis and neurosyphilis (34, 35). In the case of neuroborreliosis, intracranial lesions do not usually occur and it therefore does not represent a relevant differential diagnosis of PCNSL. Nevertheless, in patients with suspected meningeosis lymphomatosa without intracerebral lesion, differentiation of CNSL and neuroinfectious diseases solely by CXCL13 may not be sufficient and thus further diagnostics are necessary for a definite diagnosis. In addition, some malignancies such as breast, lung, and renal cancer, as well as melanoma can express CXCL13 (36–38). Similarly to our observation, particularly cerebrally metastasized breast cancer with meningeosis carcinomatosa may result in high CXCL13 CSF levels in up to 50% of cases (18), precluding a reliable differentiation from PCNSL on the basis of the CSF analysis alone.

Furthermore, CXCL13 levels cannot differentiate between primary and secondary CNS lymphoma. Thus, extensive standard staging diagnosis (i.e., computed tomography of the chest, abdomen, and pelvis, ultrasonography of the testes, and bone marrow biopsy) must still be performed to rule out systemic involvement.

In our preliminary screening tests, we identified a potential novel CSF biomarker for PCNSL, the inflammatory chemokine CXCL9. CXCL9 has been previously reported to be transcribed and translated by perivascular macrophages and pericytes in the perivascular microenvironment of CNSL, where it may support the recruitment of tumor-infiltrating lymphocytes (39). Its sensitivity and specificity (with cut-off 84: 61.5 and 87.1%, respectively), however, were considerably lower than those of CXCL13. The cut-off 36 was basically as good as 84 which means that we could not find a cut-off that was as clear-cut as it was found for CXCL13. Unlike CXCL13 (28), CXCL9 is not produced by malignant B-cells (39). Therefore, we did not observe an association with meningeosis. Moreover, CXCL9 is produced in endothelial cells in CNS in healthy individuals (40) and is upregulated in other lesional CNS diseases such as autoimmune and neuroinfectious diseases (41). This may explain why a reliable differentiation of these conditions was not always possible in our study.

Tumors such as colorectal and cholangiocarcinoma also express CXCL9 (42, 43). In our study, we also found elevated CSF-levels in a patient with metastasic NSCLC and in a melanoma patient.

Beside their diagnostic potential, CXCL13 and CXCL9 were tested for their performance as prognostic biomarkers. Both cytokines have been identified as negative predictors of survival in CNSL and other tumor types such as colorectal carcinoma (18, 44, 45). In our monocentric study, no significant cut-off for CXCL13 and CXCL9 could be calculated, probably due to the low number of CNSL patients. Since a cut-off of 200 pg/dl for CXCL13 has previously been shown to predict poor survival (18), we tested it in our population. Although lower levels showed a trend for a better survival, this difference was not statistically significant. However, further multicentric studies including larger patient cohorts should be performed to determine and validate an optimal prognostic cut-off.

Interestingly, response to therapy was accompanied by a drastic reduction of CXCL13 and CXCL9 CSF levels, indicating that both cytokines may serve as biomarkers to monitor the therapy.

In conclusion, this prospective monocentric study confirms that CXCL13 has the potential to become an essential clinical tool in the diagnosis and the disease monitoring of CNSL. Furthermore, we show that CXCL9 may serve as a potential diagnostic marker for CNSL albeit its diagnostic performance is not as good as the diagnostic performance of CXCL13.

As several other potential biomarkers in CSF for CNSL have been identified to date, it would be interesting to evaluate in future studies which combination of different biomarkers allows CNSL to be diagnosed with the highest accuracy.

## Data Availability Statement

The raw data supporting the conclusions of this article will be made available by the authors, without undue reservation.

## Ethics Statement

The studies involving human participants were reviewed and approved by Ethics Committee of the Ludwig Maximilians University, Munich, Germany. The patients/participants provided their written informed consent to participate in this study. Written informed consent was obtained from the individual(s) for the publication of any potentially identifiable images or data included in this article.

## Author Contributions

LV and UK designed the study. IM, BA, UK, and SL performed the experiments. IM, LV, MH, MD, UK, and AS were involved in recruitment of patients. MH, LV, and IM collected the clinical data. UK, IM, LV, KM, and MP did the statistical analysis. IM and LV wrote the first draft of the paper. UK, IM, KM, and MP co-wrote the manuscript. All authors discussed the results, reviewed, and commented on the manuscript.

## Conflict of Interest

The authors declare that the research was conducted in the absence of any commercial or financial relationships that could be construed as a potential conflict of interest.

## Abbreviations

CSF: cerebrospinal fluid
CNSL: cerebral lymphoma
PCNSL: primary CNS lymphoma
SCNSL: secondary CNS lymphoma
AID: autoinflammatory disease
NID: neuroinfectious Disease
PBT: primary brain tumor
SBT: secondary brain tumor.
