# Supplementary material for: CXCL13 and CXCL9 CSF Levels in Central Nervous System Lymphoma—Diagnostic, Therapeutic, and Prognostic Relevance
Source: Front Neurol. 2021 Mar 26;12:654543. doi: 10.3389/fneur.2021.654543 (PMC8032970; doi:10.3389/fneur.2021.654543)
Supplement: Supplementary file 1 [file Data_Sheet_1.docx]

**Supplemental material**

**Material and methods**

**Protein array:**

35 cytokines potentially relevant for PCNSL pathophysiology were analyzed in our custom-made protein array (B7-1, BLC, BMP 6, BMP-7, CD 40, Gro- α, IL-10, IL-4, IL-6, I-TAC, Osteopontin, SDF-1α, IL-13, IL-9, IL-7, IL-8, IL-2, IL-3, CD 27, MMP-9, ICAM-1, MCP-1, IL-18, IL-1α, GM-CSF, G-CSF, VEGF, MIP-1α, PDGF-Ra, CXCL9, IP-10, IL-6R, FAS, and TNF RII). Briefly, the CSF of all patients per group was pooled and a total of 500 µg protein per group was applied to the array membranes. The antigen-specific immunoreactivities were determined by subsequent incubation with a biotinylated antibody cocktail, horseradish peroxidase-labeled streptavidin, and chemiluminescence detection reagent. The chemiluminescence signal was detected and analyzed with a UVP ChemiDoc-IT imaging system. In order to normalize the results, the optical densities of each dot were expressed as a percentage of the average optical densities of the 6 positive controls on each array membrane. Because the protein array detects only relative expression levels and not absolute values, there is no defined lower detection limit. However, for the sake of specificity, we limited the sensitivity of the test by ignoring expression levels <5% of the positive controls. Equal to or more than 2-fold differences of the expression levels between diagnostic groups were considered relevant.

**Control patients - diagnoses**

PBT (n = 24): 10 patients with low grade glioma, 9 patients with glioblastoma multiforme, 1 patient with oligodendroglioma, 3 patients with medulloblastoma, and 1 patient with ependymoma

SBT (n = 22): 6 patients with melanoma, 5 patients with breast cancer, 8 patients with lung cancer, 1 patient with adenocarcinoma, 1 patient with acute lymphatic leukemia, and 1 patient with squamous cell carcinoma

AID (n = 29): 14 patients with multiple sclerosis, 1 patient with NMDA-receptor-positive encephalitis, 1 patient with myelitis, 1 patient with MOG-antibody-associated encephalitis, 2 patients with ADEM, 1 patient with paraneoplastic encephalitis and lung cancer, 2 patients with neuromyelitis optica, 2 patients with neurosarcoidosis, 1 patient with cerebral vasculitis, and 4 patients with encephalitis of unknown origin

NID (n = 7): 2 patients with cerebral aspergilloma, 1 patient with PML, 1 patient with cerebral toxoplasmosis, 1 patient with cerebral abscess caused by fusobacterium, 1 patient with cerebral tuberculosis, and 1 patient with bacterial endocarditis, septic embolisms and meningitis

OND (n = 17): 9 patients with stroke, 1 patient with hydrocephalus, and 7 patients with lesions of unknown origin

**Historical controls:**

101 patients with the following non-lesional neurological diagnoses that underwent a diagnostic lumbar puncture in our clinic were used as negative controls in our study: 4 patients with borreliosis, where a neuroborrreliosis or other CNS infection was ruled out; 1 patient with bilateral vestibulopathy; 6 patients with dementing syndrome; 1 patient with temporary postoperative dysarthria; 1 patient with pupillomotoric disorder; 8 patients with epilepsy; 1 patient with a mononeuropathy of the femoral nerve; 1 patient with hepatic encephalopathy; 1 patient with Herpes zoster; 1 patient with Horner’s syndrome; 1 patient with hypersomnia; 5 patients with idiopathic facial palsy; 2 patients with idiopathic intracranial hypertension; 1 patient with Parkinson syndrome; 14 patients with primary headache; 5 patients with infection-associated headache; 3 patients with motor neuron disease; 2 patients with myasthenia gravis; 1 patient with narcolepsy; 5 patients with neurodegenerative disease; 3 patients with normal pressure hydrocephalus; 1 patient with idiopathic low CSF pressure; 2 patients with sixth nerve palsy; 8 patients with polyneuropathy; 4 patients with polyradiculitis; 3 patients with monoradiculopathy; 4 patients with hypesthesia/paresthesia of unknown non-lesional origin; 7 patients with somatization disorder; 1 patient with transient ischemic attack; 1 patient with tinnitus; 1 patient with trigeminal neuralgia; 1 patient with a non-lesional cerebellar syndrome; and 1 patient with cervical spinal canal stenosis.

**Statistics.**

The statistical indication of the preference of a polynomial transformation was investigated via a closed-test procedure, as suggested by Royston and Sauerbrei. Backward elimination was combined with an adaptive algorithm, which selected the best polynomial transformation for each continuous variable in turn to identify independent prognostic factors. Bootstrap resampling was done to assess the stability of the model. To judge the prognostic quality of the final model, within different prognostic groups, expected proportions of CNSL estimated from the logistic model were compared with the observed proportions of CNSL.

The R package “optimal.cutpoints” was used in the search for the optimal cut-off and the R package “party” was used for the minimum p value approach. P value adjustment was also part of the algorithm dealing with polynomial regression modeling.

**Results**

**Logistic regression**

In 1000 bootstrap samples, CXCL13 was always included when the best multiple model was determined. In 99.2% of the cases, CXCL13 was modelled as a first-degree fractional polynomial. While CXCL9 (or any transformation) and sex were seldom included (only in 18.9% and 6.0% of the cases), age was chosen more often (in 36.0% of the models). To determine which first-degree transformation of CXCL13 was the best, univariate logistic regression solely including CXCL13 was performed. The transformations “1 / square root (CXCL13+1)” and “log(CXCL13+1)” were selected with similar frequencies (in 46.3% and 45.0% of the models, respectively). The logarithmic transformation was preferred since it was also chosen in the 155 original patients and is easier to interpret. When added to log(CXCL13+1), in 152 patients with complete data for all four variables, no further variable was significant. Again applied to all 155 patients and rounded to 2 decimal places, the odds ratio for log(CXCL13+1) was 2.693 (95% CI: 1.983; 3.658). For individual CXCL13 levels, probabilities for CNSL were predicted by the logistic regression model (Figure 3). Applying the transformation log(CXCL13+1) to the CXCL13 CSF cut-off 80 pg/ml results in 4.39, with a predicted probability of 43.5% (95% CI: 30.7; 57.3%). In the logistic model, the estimate of the intercept was – 4.612 (95% CI: – 6.377; – 3.300) and the regression coefficient for log(CXCL13+1) was 0.991 (95% CI: 0.723; 1.345).

Using grouped values of log (CXCL13+1), the proportion of observed patients with CNSL within a group was opposed to the expected proportion estimated from the logistic regression model. All expected proportions lay within the 95% CI around the observed proportions.

**Suppl. Figure 1.** 35 cytokines potentially relevant for PCNSL pathophysiology were analyzed in our custom-made protein array (s. Supplementary material). CSF from patients with primary central nervous system lymphoma (PCNSL), newly diagnosed (ND) or in relapse (R) were compared with CSF from patients with tension headache, primary brain tumor (PBT) and secondary brain tumor (SBT). Three cytokines were elevated in the CSF of PCNSL compared to the other groups: CXCL13 (red box), Il-10 (blue box) and CXCL9 (green box).

**Suppl. Figure 2.** Kaplan-Meier curves for relapse-free survival using a cut-off of 200 pg/ml for CXCL13.
