# Supplementary figures and images for: CXCL13 and CXCL9 CSF Levels in Central Nervous System Lymphoma—Diagnostic, Therapeutic, and Prognostic Relevance
Source: Front Neurol. 2021 Mar 26;12:654543. doi: 10.3389/fneur.2021.654543 (PMC8032970; doi:10.3389/fneur.2021.654543)

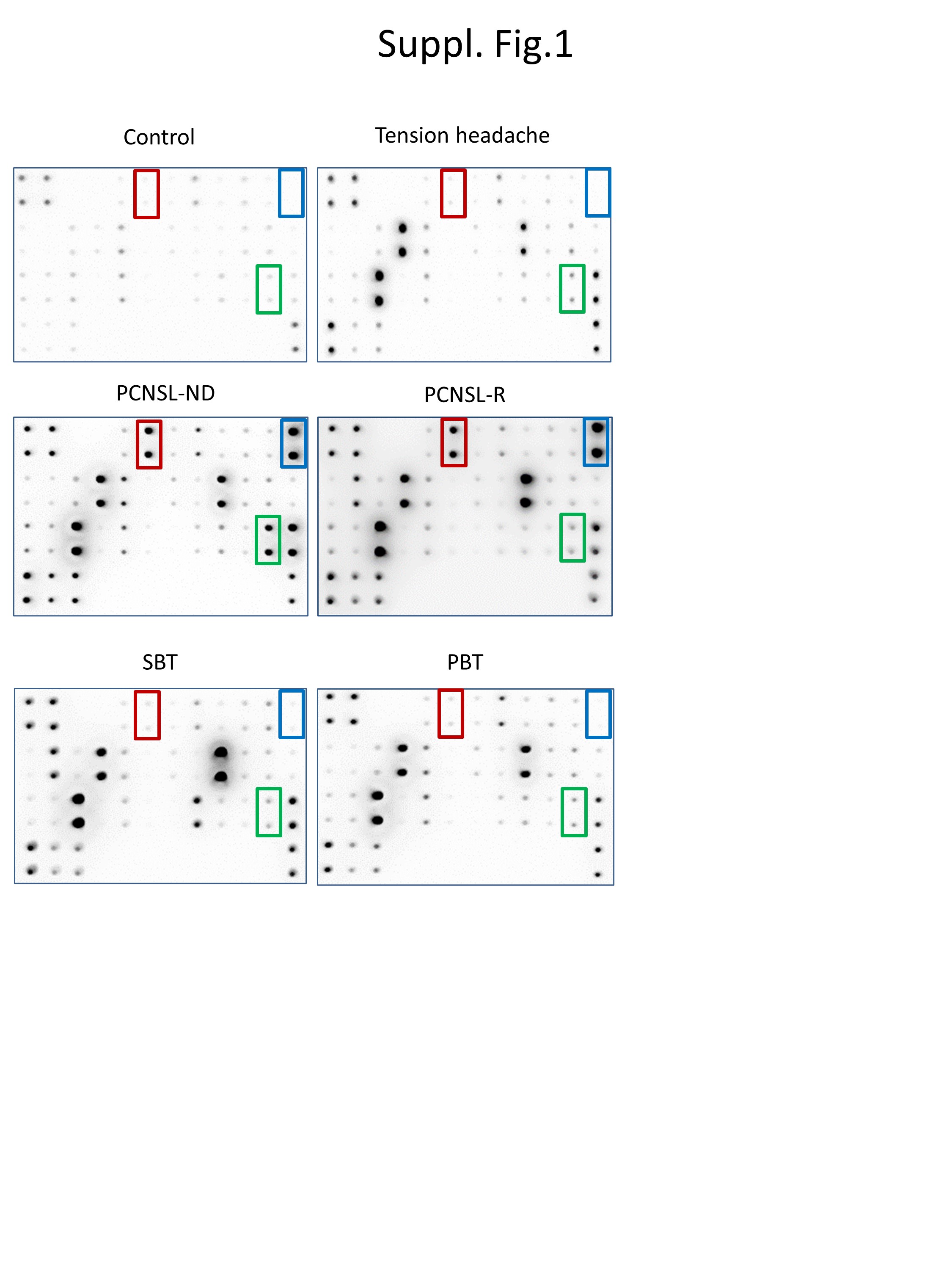

Supplement: Supplementary file 2 [file Image_1.jpeg]

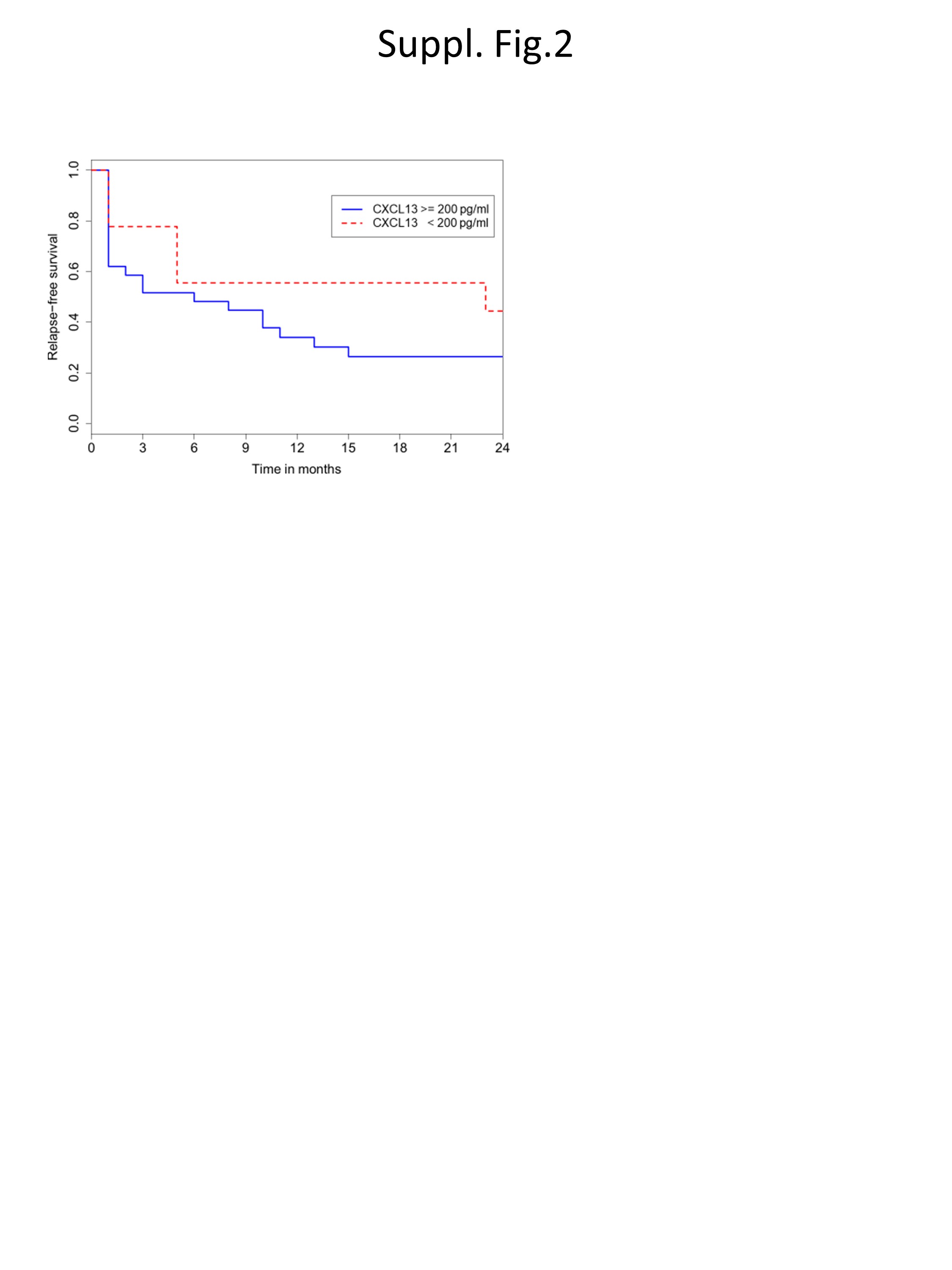

Supplement: Supplementary file 3 [file Image_2.jpeg]
